# Antiviral activity of an ACE2-Fc fusion protein against SARS-CoV-2 and its variants

**DOI:** 10.1371/journal.pone.0312402

**Published:** 2025-01-03

**Authors:** Ernesto Bermúdez-Abreut, Talia Fundora-Barrios, Diana Rosa Hernández Fernández, Enrique Noa Romero, Anitza Fraga-Quintero, Ana V. Casadesús Pazos, Briandy Fernández-Marrero, Claudia A. Plasencia Iglesias, Marilyn Clavel Pérez, Katya Sosa Aguiar, Belinda Sánchez-Ramírez, Tays Hernández

**Affiliations:** 1 Immunology and Immunotherapy Division, Center of Molecular Immunology (CIM), Havana, Cuba; 2 National Laboratory of Civil Defense (NLCD), Jamaica Highway and National Highway, San José of Lajas, Mayabeque, Cuba; University of South Florida, UNITED STATES OF AMERICA

## Abstract

SARS-CoV-2 has continued spreading around the world in recent years since the initial outbreak in 2019, frequently developing into new variants with greater human infectious capacity. SARS-CoV-2 and its mutants use the angiotensin-converting enzyme 2 (ACE2) as a cellular entry receptor, which has triggered several therapeutic strategies against COVID-19 relying on the use of ACE2 recombinant proteins as decoy receptors. In this work, we propose an ACE2 silent Fc fusion protein (ACE2-hFcLALA) as a candidate therapy against COVID-19. This fusion protein was able to block the binding of SARS-CoV-2 RBD to ACE2 receptor as measured by ELISA and flow cytometry inhibition assays. Moreover, we used classical neutralization assays and a progeny neutralization assay to show that the ACE2-hFcLALA fusion protein is capable of neutralizing the authentic virus. Additionally, we found that this fusion protein was more effective in preventing *in vitro* infection with different variants of interest (*alpha*, *beta*, *delta*, and *omicron*) compared to the D614G strain. Our results suggest the potential of this molecule to be used in both therapeutic and preventive settings against current and emerging mutants that use ACE2 as a gateway to human cells.

## Introduction

COVID-19 is one of the most devastating infectious diseases of the last hundred years, with a great impact on economic and social world dynamics, and human health. Despite the positive outcome of adopted sanitary policies and worldwide immunization against SARS-CoV-2 virus, the etiologic agent of COVID-19, the pandemic is far from over [1]. The evolution of the virus has resulted in the emergence of new variants. One of the latest is omicron, whose more concerning subvariant, XBB.1.5, has been recognized by the WHO experts as the most transmissible, with mutations in the protein spike that provoke a higher binding to the ACE2 receptor and favor the immune escape [2]. The arising strains like omicron have shown a clear reduction of protection conferred by current approved vaccines [3–5], although new variants-based boosters just start to be available [6, 7]. It should also be considered that anti-vaccine activism has gained space in some countries. This situation sabotages the gathered efforts of medical and scientific community to guarantee protection upon people immunization [8] while it is not yet well known how long lasting is the natural immunity [9, 10].

This scenario has prompted the development of therapeutic approaches targeting different infection stages. Many of them relies on blocking the gateway of the virus to the human cells, strategy led by the generation of SARS-CoV-2 specific mAbs and decoy receptors. Whereas mAbs may be susceptible to alterations in virus structure, the use of decoy receptors seems to be a readily way to neutralize this pathogen regardless the mutations that may appear.

The angiotensin-converting enzyme 2 (ACE2) is the primary receptor of SARS-CoV-2, although it has been described the existence of additional factors that can modulate or promote the entry [11–13]. ACE2 is a type I transmembrane protein, with a heterogeneous expression throughout the human body. This protein of 805 amino acids, is composed of an extracellular N terminal claw-like protease domain (PD) and a C-terminal collectrin-like domain (CLD) with a cytosolic tail [14, 15]. RBD of SARS-COV-2 directly interacts with PD, allowing the infection of host cells [16, 17]. ACE2 is also involved in the intestinal amino acids absorption [18]. However, the main physiological function is linked to its carboxypeptidase function, by converting angiotensin I (Ang I) to Ang 1–9 or Ang II to Ang I 1–7. The processing of other vasoactive peptides like neurotensin, kinetensin, and des-Arg bradykinin has been reported as well [19]. The aforementioned activities counteract those of its homolog ACE and are crucial in the regulation of renin angiotensin system (RAS). Previous investigations have demonstrated that strengthening the ACE2-Ang I (1–7)-MasR axis reduces cytokine release, conferring protection against organ injury in many human diseases, such as cardiovascular disease, chronic kidney disease, liver diseases and lung injury [20–22].

These antecedents have supported the use of ACE2 based traps as a straightforward and feasible therapeutic strategy to fight COVID-19 upon SARS-CoV-2 neutralization and possible protection from lung injury and acute respiratory distress syndrome through reestablishing the balance of RAS [23, 24]. In particular, ACE2-Fc fusion proteins may improve this receptor-based treatment by increasing the half-life and, in turn, the bioavailability of the molecule, among other advantages. From 2020 to date, many approaches depict a varied array of designs and modes of action [25–28]. While some strategies aim to reduce the potential negative impact associated to extra ACE2 peptidase activity by truncating the catalytic function, others prefer taking advantage of the regulatory role of ACE2 and maintaining the wild type form of the extracellular domain of this receptor in their constructs [29–32]. Moreover, the use of either silent or functional Fc regions has been a source of diversity for this kind of Fc fusion proteins. Several proposals rely on the use of active Fc domains, enabling virus elimination after the engagement with Fc gamma receptor on innate immune cells [29, 33]. In contrast, other groups base their traps on mutated Fc to avoid any concern regarding the possibility of enhancing the disease, through a facilitation of the virus entry, or induction of inflammation upon the stimulation of complement dependent cytotoxicity (CDC) and antibody dependent cytotoxicity (ADCC) [34, 35]. Within this group highlights the work of Tsai et al (2022), demonstrating through the use of pseudovirus assay, an improved neutralization of SARS-CoV-2 variants, including omicron, by an ACE2-Fc molecule comprising a truncated native form of the receptor (18–615) and L234A /L235A/K322A Fc (IgG1) region [32].

While the shortened version of the extracellular domain (ECD) of ACE2 is justified upon the elimination of the TRMSSP2 protease site, the utilization of full-length ECD of ACE2 grafted into LALA Fc (IgG1) region is as well a suitable choice to achieve a broad and enhanced neutralization activity against variants of concern (VOC). The use of the whole ECD of this receptor (aa 18–740) as a trap in the clinics has shown promising results and a good safety profile (ClinicalTrials.gov identifier: NCT04335136). Furthermore, a previous work reported that the ACE2-Fc containing the CLD (18–740) showed better neutralization than CLD-lacking variant (18–615), which favors its use [36].

In the present work, we propose the molecule ACE2-hFcLALA, based on ECD ACE2 (aa 18–740) and Fc (IgG1) with mutations L234A and L235A, as a candidate for the therapy against COVID-19. This molecule was able to block better the *in vitro* infection with SARS-CoV-2 VOCs (microneutralization assay with authentic virus), respect D614G strain, which suggests the potentialities of this therapeutic for being used against the current and emerging variants that maintain ACE2 as the entry portal to human cells.

## Materials and methods

### Cell lines and culture conditions

VeroE6 (ATCC CRL-1586^™^, African green monkey kidney epithelial cells), were maintained in basal growth media MEM (Gibco) supplemented with 10% fetal bovine serum (FBS; Gibco). CHOK-1 (ATCC CCL-61^™^, derived from ovary of female Chinese hamster), was cultured in protein free medium MB06. HEK293 (ATCC CRL-1573 ^™^, derived from human Embryonic Kidney) and HEK293-ACE2 (previously obtained at Center of Molecular Immunology by engineering HEK293 cells to display in cellular membrane the extracellular domain of ACE2 receptor), were cultured in DMEM/F12 supplemented with 10% FBS. All cells were grown at 37°C in a humidified atmosphere of 5% CO_2_.

### Recombinant proteins

RBD and PDL1 Fc-fusion proteins were generated at the departments of Chimeric Proteins and Protein Engineering of the Center of Molecular Immunology (CIM, Cuba), upon the coupling of either an Fc region of a human IgG1(hFc) or a murine IgG2a (mFc), to RBD of SARS-CoV-2 S protein (residues 328–533), or mouse PDL1. The irrelevant protein 6X-His tag-PDL2 was also generated at the department of Protein Engineering of CIM. 5G4 mAb (hIgG1), biosimilar candidate to Herceptin, is specific for HER2 and was generated at CIM [37].

### Generation of ACE2-hFcLALA fusion proteins

The synthetic gene of the extracellular domain (ECD) of human ACE2 (aa 18–740) (Eurofins genomics) was subcloned into pCMX plasmid bearing the gene encoding the Fc region of a human IgG1 with L234A L235A mutations, the CMV promoter and an IgG signal peptide. The transcriptional cassette coding for the fusion protein was next introduced into pL6WBlast lentiviral vector (S1 Fig) (CIGB, Cuba). Then, CHO-K1 cells were transduced with lentiviral particles to generate a stable clone secreting ACE2-hFcLALA molecule. After culture of recombinant CHO-K1 cells in MB06 medium, the supernatants were harvested, and the fusion protein was purified by using protein A affinity chromatography. The concentrations and purity of the fusion protein were determined by measuring the UV absorbance at a wavelength of 280 nm and by SDS-polyacrylamide gel electrophoresis (SDS-PAGE) (7,5%) in reducing conditions, respectively. The protein solution was 0.2μm filtered and stored at 4°C until further usage.

### Electrophoresis and Western blotting

The purified protein samples were run in 7.5% SDS-PAGE gels (Bio-Rad) under reducing conditions with 2.5% 2-mercaptoethanol and transferred to PVDF membranes. The blot was probed with a commercial ACE2 specific antibody (Novus Biological, AC18F) followed by a goat anti-mouse IgG-HRP (Cell Signaling Technologies, #7076). The Fc portion of the fusion protein was detected with a goat anti-human IgG-HRP (Cell Signaling Technologies, #32935) and the signal was visualized by enhanced chemiluminescence according to the manufacturer’s instruction (Amersham Biosciences, UK).

### Evaluation of binding of SARS-CoV-2- RBD to ACE2-hFcLALA by ELISA

#### Type I ELISA

ELISA plates were coated during 16h at 4°C with RBD-mFc fusion proteins at 5 μg/mL. Then, plates were washed with 0.05% Tween 20 in PBS (Washing Buffer) and blocked for 1h at room temperature with assay buffer (2% skim milk and 0.5% Tween 20, in PBS). Next, ACE2-hFcLALA was added at different concentrations and incubated for two hours at 37°C, followed by an incubation for 1h at 37°C with 50μL of an anti-human IgG Abs (Fc-specific) AP-conjugated (Sigma A3188). Finally, pNPP was added, and plates were incubated for 30 minutes at room temperature with light protection. The reaction was stopped using 3 M NaOH. The optical density (OD) at 405 nm was measured using a microwell reader (BioTek). The EC50 values were determined by log(agonist) vs. response nonlinear regression fit analysis (GraphPad Prism). All incubations were followed by three washing steps with washing buffer. Recombinant proteins and antibody conjugates were diluted in assay buffer.

#### Type II ELISA

ELISA plates were coated with ACE2-hFcLALA fusion protein at 5μg/ml, during 16h at 4°C. Then, the plates were washed with washing buffer and blocked for 1h at room temperature with assay buffer (4% Bovine Serum Albumin and 0.5% Tween 20, in PBS). Next, 50μL of SARS-CoV-2 Spike-His (R&D Systems, 10499) or RBD-His were added at different concentrations and incubated for 1h at 37°C. The binding of RBD proteins was detected by incubation for 1h at 37°C with 50μL of a mouse anti-His HRP-conjugated antibody (Sigma, A7058). Finally, ortophenylendiamine (OPD) peroxidase substrate (Sigma) was added, and plates were incubated for 30 minutes at room temperature with light protection. The reaction was stopped using 10 M H_2_SO_4_. The optical density (OD) at 492 nm was measured using a microwell reader (BioTek). All incubations were followed by three washing steps with washing buffer. Recombinant proteins and antibody conjugates were diluted in assay buffer.

### Evaluation of ACE2-RBD interaction inhibition by ELISA

ELISA plates were coated with ACE2-hFcLALA fusion protein at 5μg/ml, during 16h at 4°C. Then, the plates were blocked for 1h at room temperature with assay buffer (4% BSA and 0.5% Tween 20 in PBS). Serial dilutions of ACE2-hFcLALA were pre-incubated with 25ng/mL of RBD-mFc or 2μg/mL of SARS-CoV-2 Spike-His for 1 h at 37°C. The mixtures were added to the plates (50μl/well) and incubated for 1 h at 37°C. The binding of RBD proteins was detected by incubation for 1h at 37°C with 50μL of a goat anti-mouse IgG-HRP (Cell Signaling Technologies, #7076) or with mouse His specific HRP-conjugated antibody (Sigma, A7058). Finally, OPD (Sigma) was added, and plates were light protection incubated for 30 min at RT. The reaction was stopped using 10 M H_2_SO_4_. The OD at 492 nm was measured using a microwell reader (BioTek). All incubations were followed by three washing steps with washing buffer. Recombinant proteins and antibody conjugates were diluted in assay buffer.

ACE2-hFcLALA-mediated inhibition was calculated and expressed as percentage according to the next formula: Inhibition (%) = [1−(OD450nm sample/OD450nm maximal recognition)] × 100. Maximal recognition corresponds to RBD-mFc (25ng/mL) or SARS-CoV-2 Spike-His (2μg/mL).

### Inhibition of binding of RBD to ACE2 on cell membrane

VeroE6 and HEK293-ACE2 cells, cultured in DMEM containing 10% FBS, were trypsinized and washed with blocking buffer (1% BSA in PBS). The cells were incubated with a mix of serial dilutions of ACE2-hFcLALA and RBD-mFc at 100ng/mL. After 30min at 4°C, the cells were washed with blocking buffer and incubated with a mouse Fc specific FITC-conjugated antibody (Sigma, F8264) during 30min at 4°C. Cell fluorescence was monitored using a Gallios flow cytometer (Beckman-Coulter, USA). and analysed by FlowJo (v10) software. ACE2-hFcLALA-mediated inhibition was calculated and expressed as percentage according to the next formula: Inhibition (%) = [1-(% cells recognized per sample/ cells maximal recognition)] × 100. Cells maximum recognition corresponds to RBD-mFc (100ng/mL) alone.

#### MTT assay

Cell viability was assessed by MTT (Sigma-Aldrich, M5655). VeroE6 cells were seeded in 96-well plates (5×10^3^ cells/well). After reaching 60%–70% of confluency, cells were incubated with different concentrations of the recombinant protein ACE2-hFcLALA (from 6.25 μg/mL to 400 μg/mL). Untreated cells and cells treated with the human 5G4 antibody (400μg/mL) were considered as negative death controls. Doxorubicin (Sigma-Aldrich, D1515) (10μg/mL) was used as a positive death control. After 72 h, the MTT reagent was added to the cells (1 mg/ml), and 2 h later, the formazan crystals were dissolved in DMSO. Absorbance was measured at 540 nm, and background at 630 nm was subtracted. Untreated cells were considered as maximum viability control.

### Virus neutralization assay

Vero E6 cells were seeded in 96-well plates (5x10^3^ cells per well) (Costar) in MEM containing 5% FBS. 24 hours post-seeding, the cells were infected with 50MOI of the SARS-CoV-2 virus variants (D614G, Delta, Beta, Alpha, Omicron) mixed with different concentrations of ACE2-hFcLALA. After 1h of incubation at 37°C, the cells were washed with PBS and kept in MEM culture medium for 72h at 37°C and 5% CO_2_. The infectivity inhibition was determined by measuring the cellular viability of the VeroE6 cells using a colorimetric read‐out. ACE2-hFcLALA-mediated viral inhibition was calculated and expressed as percentage according to the next formula: Viral inhibition (%) = (OD samples-OD viral control)/ (OD maximal viability-OD viral control) × 100. Maximal cells viability corresponds to non-infected Vero cells.

### Virus progeny neutralization assay

Vero E6 cells were seeded in 96-well plates (5x10^3^ cells per well) (Costar) in MEM containing 5% FCS. 24 hours post-seeding, the cells were incubated with 50MOI of D614G SARS-CoV-2 virus during 1h at 37°C. Next, the cells were washed with PBS and kept in MEM culture medium with different concentrations of the recombinant protein ACE2-hFcLALA for 72h at 37°C. The infectivity inhibition was determined by measuring the cellular viability of the VeroE6 cells using a colorimetric read‐out. ACE2-hFcLALA-mediated viral inhibition was calculated and expressed as percentage according to the next formula: Viral inhibition (%) = (OD samples-OD viral control)/ (OD maximal viability-OD viral control) × 100. Maximal cells viability corresponds to non-infected Vero cells.

### Colorimetric read‐out for cellular viability determination

Supernatant of each plate was carefully removed and 100 μl of a sterile PBS solution containing 0.02% neutral red (Sigma, St. Louis, MO) was added to each well. After 1 hour of incubation at room temperature, the neutral red solution was discarded, and the cell monolayer was washed twice with sterile PBS containing 0.05% Tween 20. The PBS was carefully removed and 100 μL of a lysis solution (50 parts of absolute ethanol (Sigma, St. Louis, MO), 49 parts of MilliQ and 1 part of glacial acetic acid (Sigma)) was added to each well. Plates were incubated for 15 minutes at room temperature and then read in a spectrophotometer at 540 nm.

## Results

### Generation of ACE2-hFcLALA fusion protein

**Fig 1:**
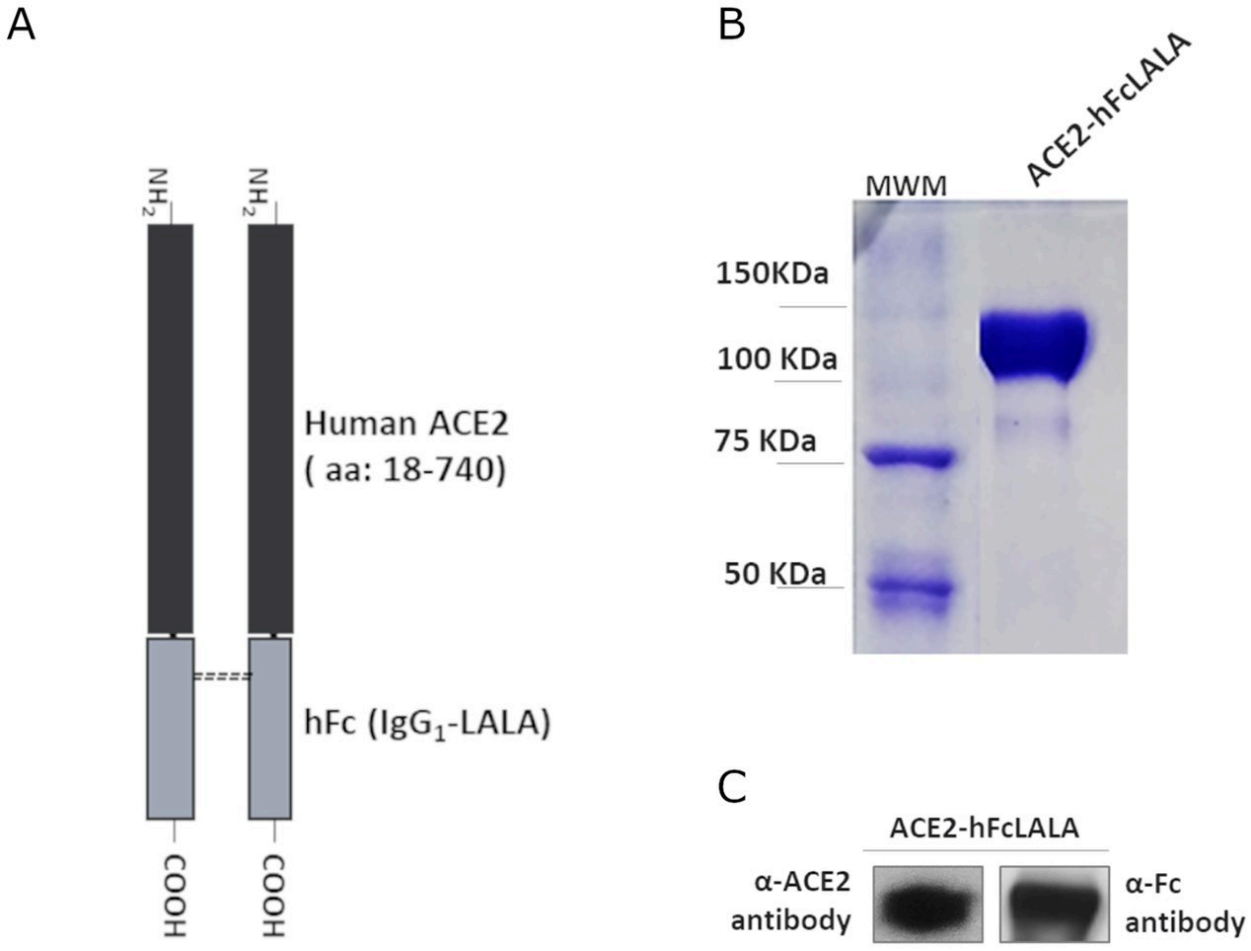
Characterization of ACE2-hFcLALA fusion protein. **(A)** Schematic representation of ACE2-hFcLALA fusion protein. Subunits of the homodimeric protein are formed by the extracellular domain of the human ACE2 fused to the Fc region of a human IgG1 with LALA mutations. **(B)** SDS-PAGE (7.5%) of purified ACE2-hFcLALA in reducing conditions. MWM, molecular weight marker (Bio-Rad, 161–0373). **(C)** Western blots analysis using antibodies specific for hACE2 (left) and human Fc fragment (right).

The ACE2-hFcLALA fusion protein was designed as a homodimer of a polypeptide consisting of the extracellular domain of human ACE2 (residues 18–740) linked to the Fc domain of human IgG1 (L234A L235A) (Fig 1A). The fusion protein was stably expressed in CHO-K1 cell line, and the recombinant protein was purified by protein A affinity chromatography. The purified ACE2-hFcLALA was evaluated by SDS-PAGE in reducing conditions, showing a band with molecular weight between 100–150 KDa, in agreement with the theoretical molecular weight for a monomeric ACE2-hFcLALA (110kDa) (Fig 1B). Both, ACE2 extracellular domain and Fc moieties were immunodetected by Western Blot (Fig 1C).

### ACE2-hFcLALA binds to SARS-CoV-2 Spike and RBD

**Fig 2:**
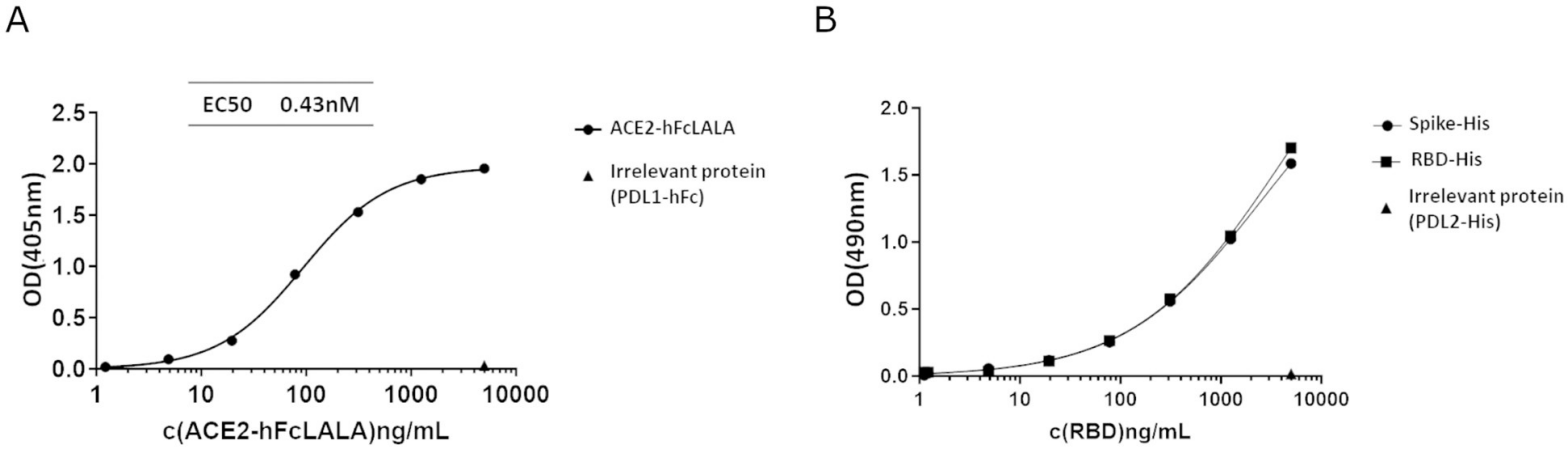
Binding of ACE2-hFcLALA fusion protein to SARS-CoV-2 RBD and Spike proteins. **(A)** ELISA plates coated with RBD-mFc fusion protein (5μg/mL) were incubated with ACE2-hFcLALA at different concentrations, followed by human Fc specific PA-conjugated antibody. PDL1-hFc was used as an irrelevant fusion protein. **(B)** ELISA plate coated with ACE2-hFcLALA (5μg/ml) were incubated with RBD-His and Spike-His at different concentrations, followed by a mouse His specific HRP-conjugated antibody. The x-axis displays the concentration of soluble purified RBD (RBD-His) or RBD domain in the context of the Spike protein. Binding was detected with pNPP **(A)** and OPD **(B)** substrates.

Next, we evaluated the binding of ACE2-hFcLALA to SARS-CoV-2 RBD-mFc adsorbed in solid phase, by ELISA. As shown in Fig 2, The ACE2-hFcLALA recognized specifically the RBD moiety (no binding to irrelevant Fc fusion protein), in a dose dependent manner and with EC50 of 0.43nM (Fig 2A). In a second assay, we studied whether the binding occurs not only to RBD alone, but to this domain in the context of the whole spike. As illustrated in Fig 2, immobilized ACE2-hFcLALA was able to specifically bind both proteins (Fig 2B).

### ACE2-hFcLALA inhibits the ACE2-RBD interaction

**Fig 3:**
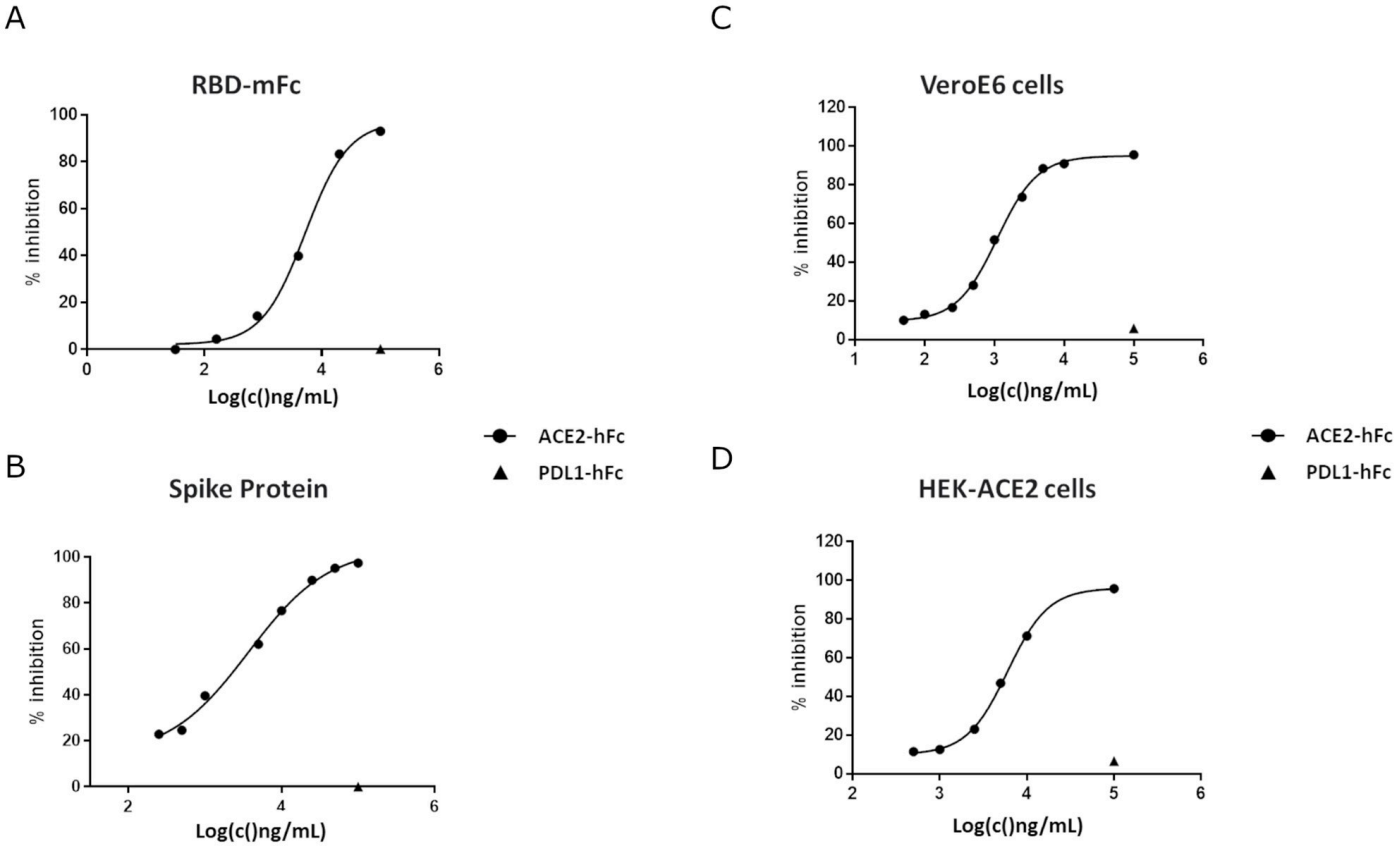
Inhibition of RBD-ACE2 interaction in vitro by ACE2-hFcLALA. ELISA plates coated with ACE2-hFcLALA (5ug/mL) were incubated with RBD-mFc (25ng/mL, **A**) or Spike (2ug/mL, **B**) premixed with ACE2-hFcLALA at different concentrations. The binding of RBD-mFc and Spike was detected with a mouse Fc specific PA-conjugated antibody/pNPP substrate and mouse HisTag specific HRP-conjugated antibody/OPD substrate, respectively. VeroE6 (**C**) and HEK293-ACE2 (**D**) cells were incubated with RBD-mFc (100ng/mL) premixed with ACE2-hFc at different concentrations. The binding of RBD-mFc was detected by flow cytometry using a mouse Fc specific FITC-conjugated antibody.

Before proving the concept of ACE2-hFcLALA as a trap of the SARS-CoV2 virus, we first evaluated the capability of its soluble form of inhibiting the RBD binding to immobilized ACE2-hFcLALA. Preincubation of ACE2-hFcLALA with SARS-CoV-2 RBD-mFc fusion protein, perfectly blocked its interaction with the purified fusion protein adsorbed in solid phase. (Fig 3A and 3B). Of note, soluble ACE2-hFcLALA was also capable of blocking the binding of the whole Spike protein to the immobilized ACE2-hFcLALA. In both cases, the specificity of the inhibition was supported by the lack of blocking activity with an irrelevant fusion protein. Then, we proceeded to evaluate whether the ACE2-hFcLALA was able to inhibit the interaction of RBD with the receptor displayed in the cell membrane (Fig 3C and 3D). In this experiment, it was observed that ACE2 fusion protein inhibits, in a dose-dependent manner, the interaction of RBD to VeroE6 monkey cells that express an ACE2 receptor highly homologous to that of humans [38, 39] (Fig 3C). Furthermore, the ACE2-hFcLALA was able to prevent the binding of RBD to HEK293 cells engineered to express the extracellular domain of human ACE2 in the cell membrane. (Fig 3D). Taken together, these evidences suggest the ability of the newly generated fusion protein to capture RBD and impede its binding to the membrane receptor.

### ACE2-hFcLALA prevents the infection of VeroE6 cells with SARS-CoV-2

**Fig 4:**
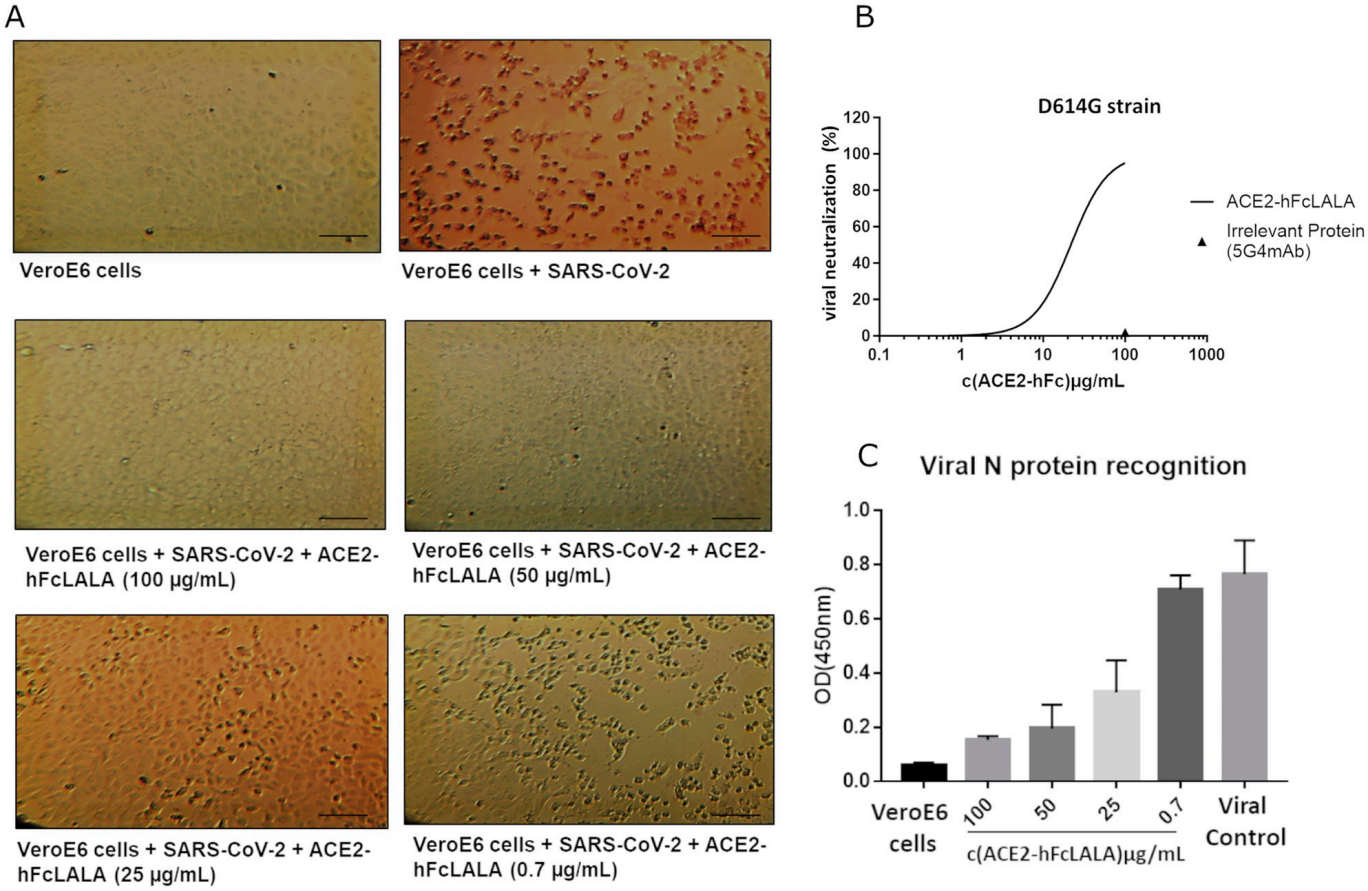
Neutralization of SARS-CoV-2 virus by ACE2-hFcLALA. VeroE6 cells were infected with a mixture of the D614G SARS-CoV-2 virus and ACE2-hFcLALA at different concentrations. After 1h at 37°C of contact with the mixture, the cells were PBS washed and kept in culture for 72h at 37°C. (**A**) Images of VeroE6 cells alone, virus infected, and infected with a mixture of viruses and different concentrations of ACE2-hFc. Magnification 10X. Scale bar 100 μm (**B**) The infectivity inhibition was determined by measuring the cellular viability of the VeroE6 cells. The human 5G4 antibody was used as infectivity inhibition negative control. (**C**) Recognition of SARS-CoV-2 N proteins using two different epitopes specific monoclonal antibodies against this protein.

To investigate whether ACE2-hFcLALA can hinder the infection with SARS-CoV-2 D614G, a conventional neutralization assay was performed. Herein, Vero E6 cells were challenged with the virus alone, or mixtures of the virus and different concentrations of the fusion protein. Non-cytotoxic direct effect of the ACE2-hFcLALA was observed on VeroE6 cells (S2 Fig). As illustrated in Fig 4, the incubation of ACE2-hFcLALA with the SARS-CoV-2 strongly protected VeroE6 cells from viral infection (IC50 of 21.8μg/mL, 99.6 nM), as assessment of cell viability (Fig 4B). The intact monolayer formed by VeroE6 cells in culture, was used as a negative control of viral infection. The positive control of viral infection was VeroE6 cells incubated with SARS-CoV-2 alone, resulting in the total rupture of the cell monolayer (Fig 4A). In agreement with that result, it was observed a decrease in the content of SARS-CoV-2 N protein, in the supernatant of the samples treated with the virus pre-mixed with ACE2-hFcLALA (Fig 4C). This evidence demonstrates the ability of ACE2-hFcLALA fusion protein to inhibit viral infection by SARS-CoV-2.

### ACE2-hFcLALA inhibits the infection of SARS-CoV-2 virus progeny

**Fig 5:**
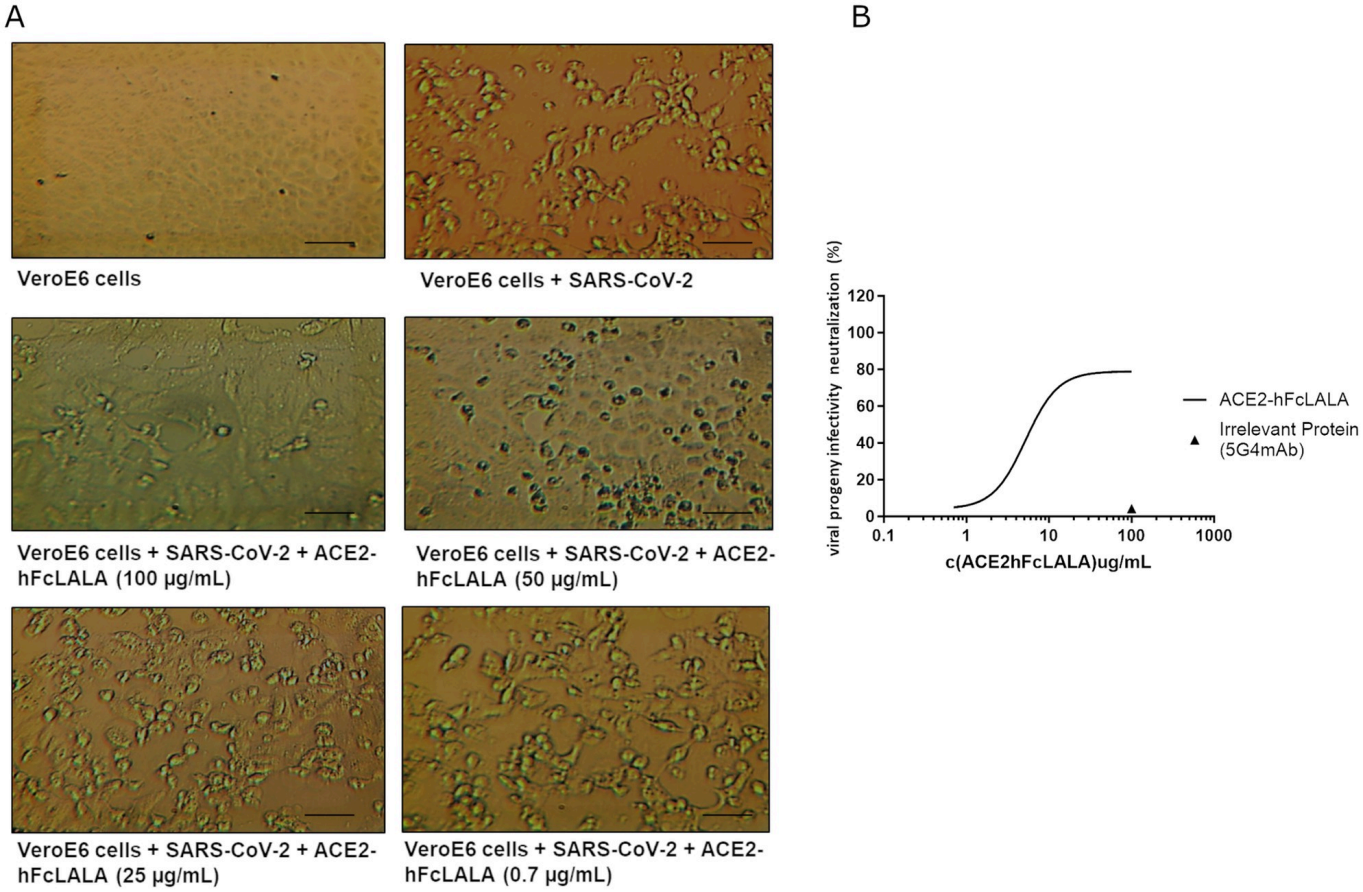
Neutralization of SARS-CoV-2 virus progeny by ACE2-hFcLALA. VeroE6 cells were infected with a single-round infection of D614G SARS-CoV-2 virus during 1h at 37°C. Then, the cells were PBS washed and kept in culture with different concentrations of the recombinant protein ACE2-hFcLALA for 72h at 37°C. (**A**) Images of VeroE6 cells alone, virus infected and infected and kept in culture with different concentrations of ACE2-hFc. Magnification 10X. Scale bar 100 μm (**B**) The infectivity inhibition was determined by measuring the cellular viability of the VeroE6 cells. The human 5G4 antibody was used as infectivity inhibition negative control.

In another experiment, we examined the capability of ACE2-hFcLALA of tackling the infection mediated by SARS-CoV-2 progeny. VeroE6 cells pre-challenged with a single-round infection with D614G SARS-CoV-2 for one hour, were washed and subsequently kept in culture for 96 hours with different concentrations of ACE2-hFcLALA. The incubation of VeroE6 cells with ACE2 fusion protein protected uninfected cells (IC50 of 5.3μg/mL or 24.2nM) from viral progeny infection (Fig 5A and 5B). Noteworthy, in this scenario the protection is not complete because during the first 1-hour encounter with the virus, about 20% of cell get infected (Fig 5B).

### ACE2-hFcLALA inhibits the infection of SARS-CoV-2 authentic virus variants

**Fig 6:**
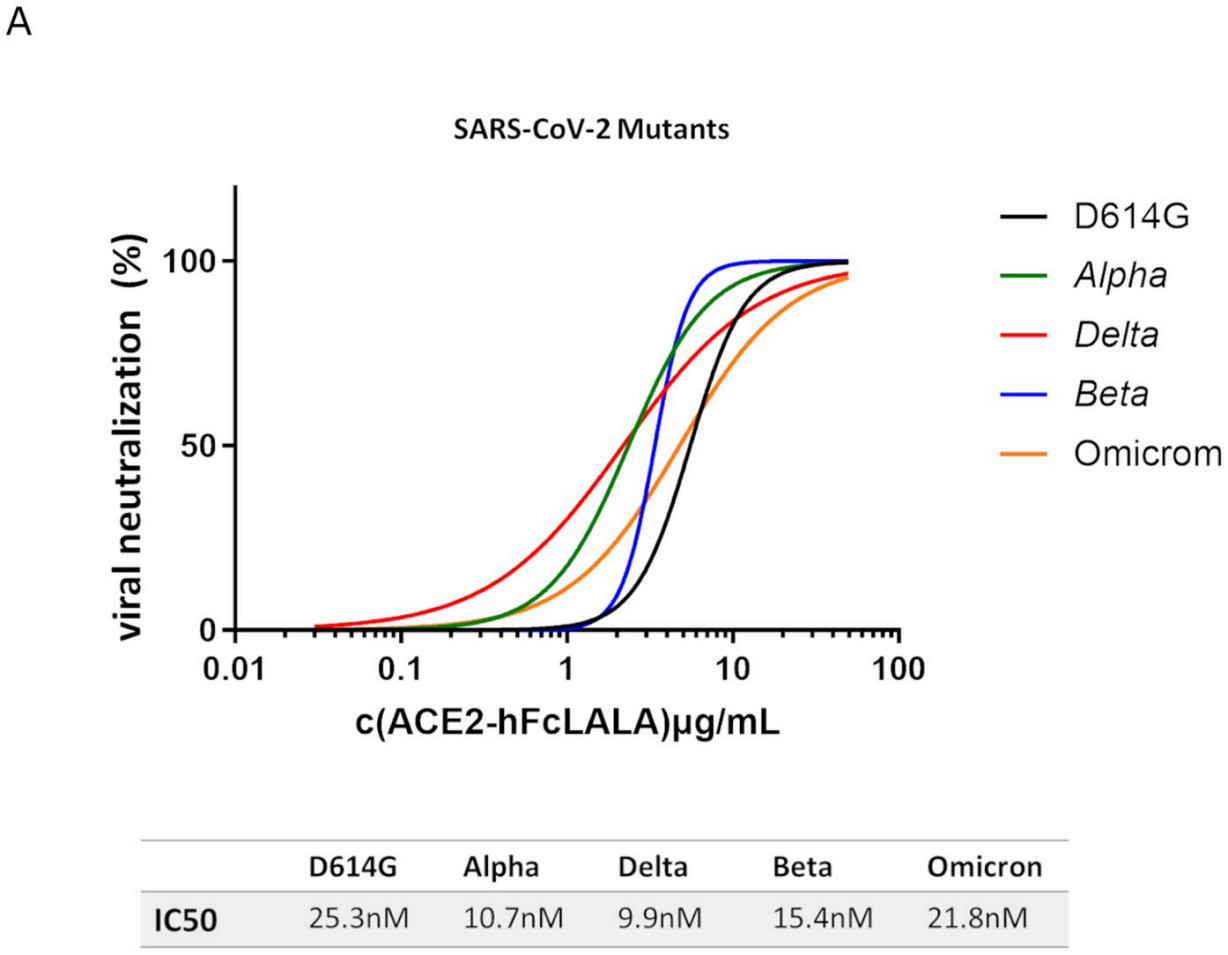
Neutralization of SARS-CoV-2 virus variants by ACE2-hFcLALA. VeroE6 cells were infected with a mixture of each of the five mutated SARS-CoV-2 virus variants and different concentrations of the recombinant protein ACE2-hFcLALA. After 1h at 37°C in contact with the mixture, the cells were PBS washed and kept in culture for 72h at 37°C. The infectivity inhibition was determined by measuring the cellular viability of the VeroE6 cells. The human 5G4 antibody was used as infectivity inhibition negative control.

Finally, we conducted a series of viral inhibition assays using Alpha, Beta, Delta, Omicron and D614G mutated SARS-CoV-2 virus variants (Fig 6). VeroE6 cells were infected with a mixture of each of the five mutated SARS-CoV-2 variants and different concentrations of the recombinant ACE2-hFcLALA. After 1h contact with the mixture, the cells were washed and kept in culture for 72h. The infectivity inhibition was determined by measuring the cellular viability of the VeroE6 cells, using non-treated cells as a reference. ACE2-hFcLALA fusion protein was able to inhibit all SARS-CoV-2 variants infection. The IC50 (nM) values for D614G, *Delta*, *Alpha*, *Beta* and *Omicron* variants were 25.3, 10.7, 9.9, 15.4 and 21.8 nM, respectively. All the variants were easier to inhibit respect the D614G, being the *Delta* the one with the highest sensitivity to be blocked.

## Discussion

The effectiveness of mAbs and profilactic vaccines against SARS-CoV-2 may be fundamentally threatened by the appearance of multiple variants of the virus, with the RBD of the SARS-CoV-2 being a key site for immune escape mutations [3, 40–45] and enhanced binding to ACE2 receptor on the host cells [43]. In this sense, finding a treatment that can confront the generation of virus evolving variants remains a need.

In this work, we validate the concept of ACE2-human Fc decoy receptors as promising tools to be used against possible new SARS-CoV-2 variants that may evade vaccination, mAbs and other treatments. Here, we demonstrated that the *in vitro* neutralizing capability of ACE2-hFcLALA against the mutants *Alpha*, *Beta*, *Delta* and *Omicron* was higher than that against the D614G, a widespread established variant during the early phase of pandemics, with similar morphology and in vitro neutralization properties [46, 47] to ancestral wild-type virus, enabling its use as a control. We also found that the *Delta* variant was the most sensitive to being blocked, followed by the *Alpha* variant. Previous bioinformatic studies reported that the *Delta* and *Alpha* variants showed higher levels of affinity for the ACE2 receptor with the consequent greater stability of the interaction, compared to the wild-type variant [48–50], which is consistent with the viral inhibition results obtained in this work.

Moreover, our result confirms the findings of Tsai et al. (2022) who showed that a truncated form (aa18-615) of ACE2 ECD fused to Fc LALA/K322A exhibited an increased blocking potency for SARS-CoV-2 pseudotyped virus mutants respect the D614G variant [32]. Like us, Tsai also found that after comparing *Alpha*, *Beta*, *Delta*, *Omicron and* wild type variants, the *Delta* variant was the most sensitive to being blocked, followed by the *Alpha* variant. In contrast with Tsai’s works that used pseudovirus assay, we accomplished the neutralization experiments by using authentic virus, which enables a more accurate assessment of the actual neutralizing capacity of the fusion protein [51]. In this sense, while a pseudovirus systems expressing unique S proteins is a suitable method for studying viral strains and their variants, it’s important to note that the distribution and density of such proteins in these artificial particles may not accurately reflect their levels of expression and epitopes in natural viruses [52]. In addition, despite pseudovirus assays reproduce the process of binding to receptor and further cell entry, they are generally limited by their inability to support viral replication [53]. In our work, viral replicative capacity is essential because we also studied the ACE2-hFcLALA-mediated inhibition of the infectivity of viral progeny, which depends on the virus ability to replicate. Our results showed the blocking capacity of the ACE2-FcLALA over SARS-CoV-2 progeny virions, an important element not documented in previous works. This is of paramount importance as it is well known that the number of progeny virions produced is crucial in the success of a viral infection at the cellular level [54]. Other work has also reported the blocking effect of ACE2-Fc bearing LALA mutations, by using active virus, but was limited to *delta* and *omicron* variants [55].

In particular, the performed classic neutralization assays suggest the potential efficacy in the preventive setting, which supports the use of this trap in SARS-CoV-2 exposed personal as early predicted during the first moments of COVID19 pandemic [56]. On the other hand, the results from the progeny inhibition assays indicate a likely protective action of ACE2-FcLALA, even in cells previously exposed to the virus, and in turn, in a therapeutic scenario.

This represents an advantage over several mAbs used for COVID-19 therapy, whose efficacy against the different variants of the virus has been reduced, despite their moderate efficacy and good safety profiles in the clinics during the first outbreak waves [57]. Numerous *in vitro* studies have investigated the impact of mutations of SARS-CoV-2 variants used in this work on resistance to treatment with SARS-CoV-2 Spike specific mAbs. For example, Etesevimab has shown a 5-fold decreased efficacy against the Alpha and Omicron variants, while Bamlanivimab and Regdanvimab, also showed a reduced effectiveness against the Delta mutant. In the case of the Casirivimab, a decreased efficacy against the Alpha, Beta Delta, Omicron variants was observed as well [58–67].

Here, we are committed to a full-length extracellular domain (18-740aa) of ACE2 in our fusion protein, instead of a truncated form of the ECD of this receptor. A previous work of Li et al. (2020) demonstrated that the use of the whole ACE2 ECD confers more than 20-fold improvement in potency than the short ACE2 (18-615aa) variant in a similar Fc fusion protein. The reason for this improvement is not very clear, but it has been speculated that it is possible the CLD domain, included only in the larger version, stabilizes an orientation of the ACE2 enzymatic domain favorable to S-protein binding, or that the CLD has an independent anti-SARS-CoV-2 activity [36].

ACE2 molecules fused to Fc domains of human immunoglobulins have taken advantage of Fc region to extend the half-life time of these recombinant proteins upon interaction with FcRn [68, 69]. In our opinion, the presence of wild type Fc sequences responsible for binding to FcRn in this type of molecule is sufficient to increase the persistence of the molecule in circulation but it would be suitable to be cautious in prolonging excessively the life time circulation to avoid detrimental effects. However, in a Phase 1 clinical trial (ClinicalTrials.gov identifier: NCT04583228), HLX71, a recombinant human angiotensin-converting enzyme 2 (ACE2)-Fc fusion protein developed for COVID-19, showed good safety and tolerability in healthy adult subjects. This indicates that this type of construct is safe (https://www1.hkexnews.hk/listedco/listconews/sehk/2022/0314/2022031401097.pdf).

In this work, although we did not measure the catalytic activity of our fusion protein, we addressed the strategy of using a molecule designed to display an ACE2 intact catalytic activity. It has been widely recognized the therapeutic benefits of these decoy receptors beyond direct binding to the viral spike protein, which has been directly associated to enzymatic action of ACE2 [70]. There has been clearly defined two opposed positions regarding the use of functional ACE2 in this Fc fusion proteins. The discrepancy relies on whether sACE2-catalyzed turnover of vasoconstrictive and proinflammatory peptides will confer therapeutic benefits or whether it is a safety concern. Several groups have eliminated the catalytic activity when developing decoy receptor candidates, preventing ill-defined risks of adverse hypotension [29, 30, 71]. Even though, the administration of functional ACE-2 based biologics has shown to have a good safety profile in a study comprising repeated doses of ACE2-Fc in mice, and soluble ACE2 in Phase I and Phase II clinical trials for human pulmonary arterial hypertension and acute respiratory distress syndrome (ARDS) (ClinicalTrials.gov identifier: NCT04335136 and NCT04583228).

While coupling of ACE2 to the Fc region enables the biological effector functions of this domain to be exploited [29], there is a current discussion about the benefit/risk of using ACE2-Fc fusion proteins. The main issue under discussion is the potential threat of ADE. This effect can be mainly caused by two mechanisms. One of them promotes the viral infection through Fc-mediated interaction with FcγR present on certain effector cells (i.e., monocytes and macrophages). The other one leads to elevated inflammation upon the activation of FcγR receptor bearing cells, that may worsen disease. For these reasons, in our work we decided for a silent Fc (LALA mutated) just to take advantage of the Fc features related to increased avidity and half life time, besides the manufacturability gains in terms of higher expression and easier purification [72]. Also, the approval of many Fc fusion proteins by regulatory agencies and their application in the clinics for the treatment of different diseases has demonstrated and indicates the safety and tolerability of this Fc tag in patients with various conditions [73].

In summary, our data support ACE2-FcLALA fusion protein as a candidate to be used to protect against any SARS-CoV-2 variant with equal or greater affinity for the ACE2 receptor, or theoretically against even any virus that uses this receptor as an entry portal to the organism. This molecule could be an alternative to consider in health systems to immediately using as a treatment or prophylactically in possible future outbreaks of new SARS-CoV-2 variants or new viruses that use ACE2 as a gateway.

## Supporting information

S1 Fig (A) pL6WBlast-ACE2-hFcLALA lentiviral vector map. (B) DNA sequence encoding the extracellular domain (from amino acid 18 to amino acid 740) of the human ACE2 fused to human Fc (L234A, L235A mutations).
(DOCX)

S2 Fig Non-cytotoxic direct effect of the ACE2-hFcLALA. VeroE6 cells were treated with different concentrations of the recombinant protein ACE2-hFcLALA and then incubated in culture for 72 hours at 37°C. Cellular viability was assessed using the MTT method. Doxorubicin (DOXO, 10 μg/mL) served as the positive control for cell death, while the human 5G4 antibody (400μg/mL) was used as the negative control. Differences among means were analyzed using one-way ANOVA and Dunnett’s test for multiple comparisons. Significant differences are represented as ***p < 0.001.
(DOCX)

S1 Raw images (PDF)
