# Supplementary material for: Antiviral activity of an ACE2-Fc fusion protein against SARS-CoV-2 and its variants
Source: PLoS One. 2025 Jan 3;20(1):e0312402. doi: 10.1371/journal.pone.0312402 (PMC11698409; doi:10.1371/journal.pone.0312402)
Supplement: S1 Fig — (A) pL6WBlast-ACE2-hFcLALA lentiviral vector map. (B) DNA sequence encoding the extracellular domain (from amino acid 18 to amino acid 740) of the human ACE2 fused to human Fc (L234A, L235A mutations). (DOCX) [file pone.0312402.s001.docx]

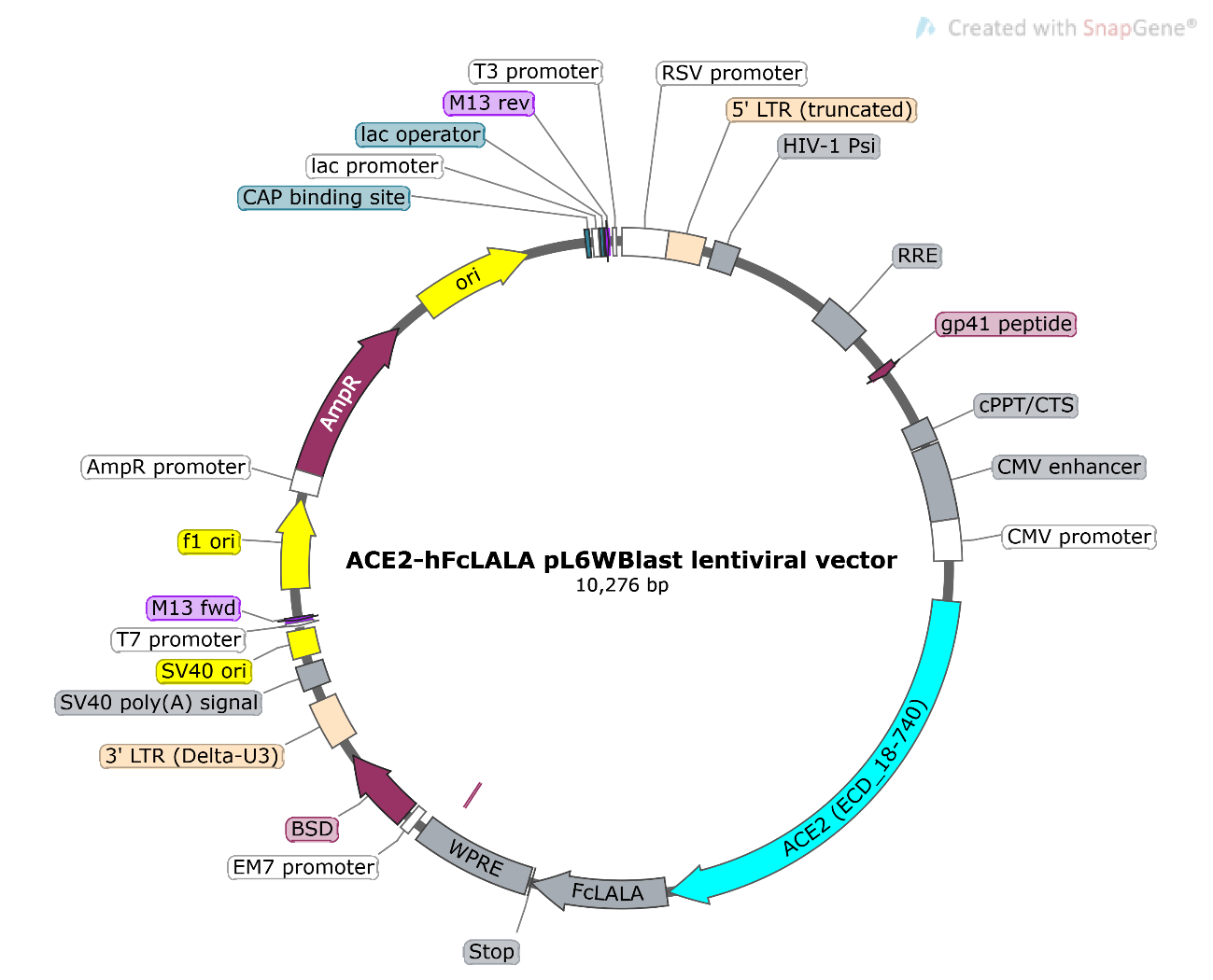


**B**

**A**

>ACE2-hFcLALA sequence

cagagcaccattgaggaacaggcaaagacattcctggacaaatttaaccacgaagcagaggacctcttctaccagtcaagcctcgccagttggaactacaacactaacattaccgaagagaatgtgcaaaacatgaataacgctggtgataaatggtcagcatttctgaaagagcagtctacactggcccagatgtatcctcttcaagagatccagaatctgacggtgaagctccaactgcaagctctccagcagaatggctccagtgtgctgagcgaggacaagtcaaaacggctgaacactatcctgaataccatgtctaccatttactccactggtaaggtctgtaatcccgataatcctcaggaatgtttgctccttgaaccagggctgaacgagatcatggccaatagccttgactacaacgagaggctctgggcctgggagtcttggagatcagaggtagggaagcagcttagacccctgtacgaggagtatgtggtcctcaagaacgaaatggcccgagccaaccactacgaggattacggcgattactggaggggagactatgaagtaaacggcgttgacggctatgactacagtcgcgggcaactgattgaagatgtcgaacacaccttcgaagagatcaaaccactgtatgagcatctgcatgcctatgtgagggccaaactcatgaatgcttatccctcctatatctctccaattgggtgtctgcccgcacatctgttgggagatatgtggggacgtttctggactaatctctactccctgacagtgccttttggccagaagcccaacattgatgtcaccgatgctatggtggatcaggcatgggatgcccaaaggatctttaaggaagccgagaaattctttgtgtctgttggactgcctaacatgactcagggattctgggagaactccatgctgaccgaccccggaaatgtccagaaggccgtttgtcaccccacagcttgggatctcgggaagggtgacttccgcatactgatgtgcaccaaagtgactatggacgatttcctgacagcccatcacgaaatgggtcacattcagtacgatatggcttatgccgcccagccatttctgctgcggaacggtgctaatgagggctttcatgaagctgtgggcgagattatgagcctgtccgctgctacacctaaacacctgaagagtatcggactgctgagtcccgactttcaggaggataatgagacagagatcaacttcttgcttaaacaagccttgacaatcgttggcacacttccctttacgtacatgctggagaaatggcgatggatggtctttaagggagagatccccaaagatcagtggatgaagaaatggtgggagatgaaaagggaaatagtcggggtggtagaaccagtgcctcacgacgaaacctattgcgaccctgctagcctgttccacgtgagcaatgactacagtttcatccggtactatacccgtaccttgtaccagtttcagtttcaggaagccctttgccaagcagctaagcatgaagggccattgcataagtgcgacatatccaatagcacggaggccggtcagaagctgttcaatatgctcagactgggcaaaagcgaaccctggactctcgcactggaaaacgtggtaggcgccaagaacatgaacgttcgtcctctgctgaactacttcgaacccttgttcacttggctgaaggatcagaacaagaactcctttgttgggtggtctacagactggtccccttatgccgaccagtctatcaaggtccgcatttctctgaagtccgctcttggcgacaaagcatacgagtggaacgataatgaaatgtacctgttcagatcatctgtcgcatatgctatgaggcagtatttcctgaaggtgaagaatcagatgatcctgtttggagaggaggacgtccgggtggcaaaccttaagccacgcatctcctttaacttcttcgtgactgctcccaagaatgtaagcgacattatcccaagaaccgaggttgagaaggcaatacgaatgtctcgatcacgcatcaatgatgcctttcggttgaatgacaacagtttggagttcctcggcattcagccaaccttgggtccacccaatcaacctcctgtgtccgcggccgctagcgacaaaactcacacatgcccaccgtgcccagcacctgaagccgcggggggaccgtcagtcttcctcttccccccaaaacccaaggacaccctcatgatctcccggacccctgaggtcacatgcgtggtggtggacgtgagccacgaagaccctgaggtcaagttcaactggtacgtggacggcgtggaggtgcataatgccaagacaaagccgcgggaggagcagtacaacagcacgtaccgtgtggtcagcgtcctcaccgtcctgcaccaggactggctgaatggcaaggagtacaagtgcaaggtctccaacaaagccctcccagcccccatcgagaaaaccatctccaaagccaaagggcagccccgagaaccacaggtgtacaccctgcccccatcccgggaggaggtgaccaagaaccaggtcagcctgacctgcctggtcaaaggcttctatcccagcgacatcgccgtggagtgggagagcaatgggcagccggagaacaactacaagaccacgcctcccgtgctggactccgacggctccttcttcctctatagcaagctcaccgtggacaagagcaggtggcagcaggggaacgtcttctcatgctccgtgatgcatgaggctctgcacaaccactacacgcagaagagcctctccctgtccccgggtaaa

**S1 Fig.**
