# Supplementary figures and images for: Antiviral activity of an ACE2-Fc fusion protein against SARS-CoV-2 and its variants
Source: PLoS One. 2025 Jan 3;20(1):e0312402. doi: 10.1371/journal.pone.0312402 (PMC11698409; doi:10.1371/journal.pone.0312402)

**
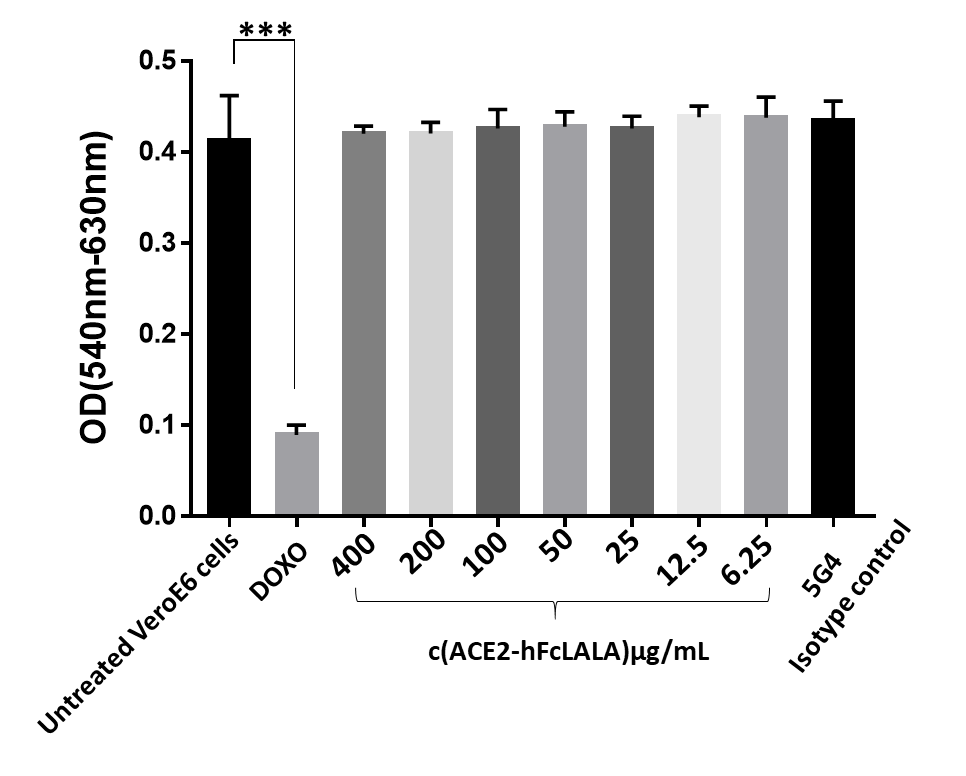
**

**S2 Fig**.

Supplement: S2 Fig — VeroE6 cells were treated with different concentrations of the recombinant protein ACE2-hFcLALA and then incubated in culture for 72 hours at 37°C. Cellular viability was assessed using the MTT method. Doxorubicin (DOXO, 10 μg/mL) served as the positive control for cell death, while the human 5G4 antibody (400μg/mL) was used as the negative control. Differences among means were analyzed using one-way ANOVA and Dunnett’s test for multiple comparisons. Significant differences are represented as ***p < 0.001. (DOCX) [file pone.0312402.s002.docx]
