# Supplementary material for: Antiviral activity of an ACE2-Fc fusion protein against SARS-CoV-2 and its variants
Source: PLoS One. 2025 Jan 3;20(1):e0312402. doi: 10.1371/journal.pone.0312402 (PMC11698409; doi:10.1371/journal.pone.0312402)
Supplement: S1 Raw images — (PDF) [file pone.0312402.s003.pdf]

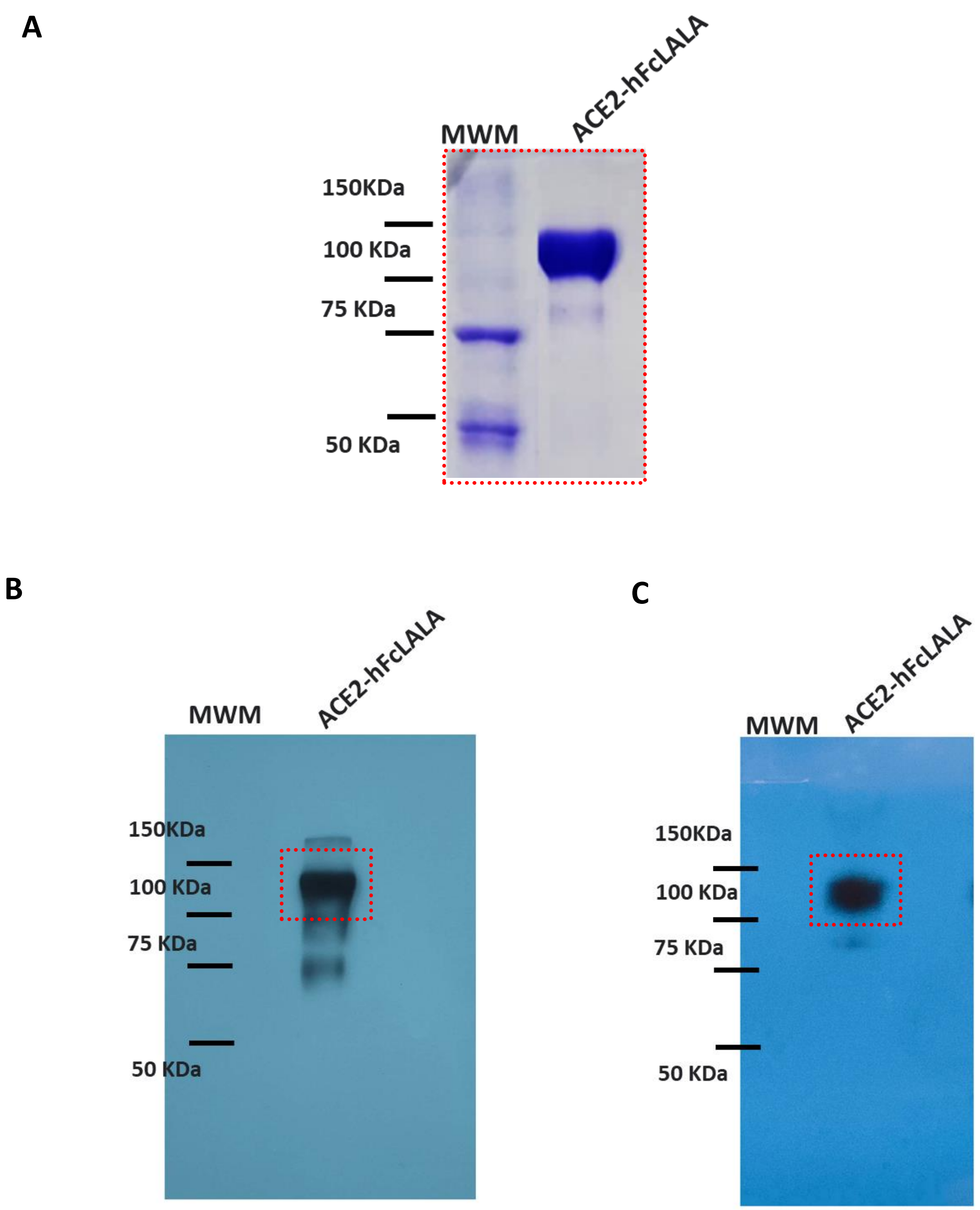

Picture of the original SDS-PAGE gel (7.5%) of the purified ACE2-hFcLALA in reducing conditions (A). Pictures of the Lumi-Film Chemiluminescent Detection Films (11666916001, Sigma) of Western Blotting with an antibody specific for the human Fc region (B) or with an antibody specific for the ACE2 receptor (C). The red dashed squares highlight the part of the original images that was used to generate the corresponding figures (see Figure 1C). MWM, molecular weight marker (Bio-Rad, 161-0373).
